# Comparison of the Efficacy of Two Elastic Bandages for Forearm Hematoma After Transradial Coronary Intervention

**DOI:** 10.3389/fsurg.2021.709489

**Published:** 2021-09-16

**Authors:** Lei An, Wei-liang Du, Xiao-Ning Yang, Chun-Yan Zhang, Zeng-Ming Xue

**Affiliations:** ^1^Department of Cardiology, Langfang People Hospital, Langfang, China; ^2^Department of Orthopedics, Langfang People Hospital, Langfang, China

**Keywords:** transradial coronary intervention, subcutaneous hematoma, elastic bandage, thrombosis, nylexorgrip elastic bandage

## Abstract

**Background:** This study compares the efficacy of two elastic bandages in treating forearm hematoma after transradial coronary intervention.

**Methods:** A total of 60 patients with moderate or severe forearm hematoma following transradial coronary intervention were enrolled in this study. They were randomly divided into two groups, as follows: an Idealast-haft elastic bandage group (the observation group) and a control group. The patients in the Idealast-haft elastic bandage group received compression bandaging with Idealast-haft elastic bandages and the patients in the control group received compression bandaging with Nylexorgrip elastic bandages. Observation indexes related to, for example, forearm pain, arterial pulsation, blistering, skin color, and hemostasis time were compared between the two groups.

**Results:** The results revealed that the times taken for pain disappearance, arterial pulse recovery, blister disappearance, skin color recovery, and compression hemostasis were significantly shorter in the Idealast-haft elastic bandage group than in the control group, and the differences were statistically significant (*P* < 0.05). The hematoma range and the arm circumference at the severest part of the hematoma decreased faster in the observation group than in the control group, and the differences were statistically significant (*P* < 0.05).

**Conclusion:** The Idealast-haft elastic bandage is more effective than the Nylexorgrip elastic bandage in patients with forearm hematoma following transradial coronary intervention and should therefore be used in such cases.

## Background

Transradial coronary intervention for diagnosis and treatment is a milestone in the history of interventional cardiology (1, 2). The radial artery approach is superficial and easily compressed and fixed; in addition, it forms a double blood supply with the ulnar artery. The many advantages of transradial intervention include the following: few complications, small trauma of the puncture site, no need for long-term bed rest, improved patient comfort, slight postoperative nursing workload, and short hospital stay (3, 4). The promotion of this technology over the last 10 years has given it global popularity. In 2017, the total number of coronary intervention cases in China exceeded 750,000. The transradial technique currently accounts for more than 80% of interventions in many medical centers in China. Although the radial artery approach has many advantages, the popularity of this technique means that the associated complications cannot be ignored (5, 6).

As the proportion of patients who undergo transradial coronary intervention has increased, postoperative complications have become more common. Forearm hematoma is the most common complication following transradial intervention, with an incidence of ~1–2%. Early detection and the use of appropriate procedures are key to preventing serious adverse events (7–9).

## Methods

### General Data

This study included patients with coronary heart disease who were admitted to our department from March 2016 to January 2018. All patients had moderate to severe forearm hematoma after coronary intervention (see Table 1). They were randomly divided into two groups, an Idealast-haft elastic bandage group (the observation group) and a control group. The patients in the Idealast-haft elastic bandage group received compression bandaging with Idealast-haft elastic bandages and the patients in the control group received compression bandaging with Nylexorgrip elastic bandages. The study was approved by the Ethics Committee of our hospital, and all patients provided signed informed consent.

**Table 1:** The hematoma classification.

**Grade**	**Scope**	**Pain**	**Blisters**	**Color of skin**	**Peripheral sensory**	**Pulse**
Mild	The range of the skin bulge is <1/3 of the forearm	Grade 1 or below	None	Normal	Normal	Normal
Moderate	The range of skin swelling reaches 1/3–1/2 of the forearm	Grade 1–2	Occasional blisters	Bruises	Normal	A little weak
Severe	The skin swelling extends over 1/2 of the forearm	Grade 2 or above	Have blisters	Cyanosis	Numb	Weaken or disappear

### Inclusion and Exclusion Criteria

The inclusion criteria for patients were as follows: (1) transradial coronary intervention by the same operator; (2) moderate or severe forearm hematoma (as defined in Table 1) after the operation; and (3) an age of ≥18 years. The exclusion criteria for patients were as follows: (1) negative Allen's test result; (2) history of radial artery puncture within half a year; and (3) complicated degeneration of the vein bridge.

### Therapeutic Methods

After the interventional therapy, the skin around the radial artery puncture point was sterilized and dried, the sheath tube was withdrawn, and local compression hemostasis was performed with a pressure apparatus. Patients with forearm hematoma during and after the operation were selected. With the local bleeding site as the midpoint, compression hemostasis with elastic bandages was performed at two sites, ~2 cm from the proximal and distal ends of the hematoma. Patients in the Idealast-haft elastic bandage group were treated with Idealast-haft elastic bandages, and patients in the control group were treated with Nylexorgrip elastic bandages. The pressure of the elastic bandages was maintained within the levels in which the pulsation of the radial artery of the affected limb could be felt, and the patients were monitored for the presence of ischemic symptoms. The elastic bandaging was relaxed every 2 h according to the condition of the subcutaneous hematoma and the chief complaint and symptoms of the patient; this adjustment continued until the bandages were removed.

### The Main Observation Indexes

In the present study, the main observation indexes included: (a) the speed of reduction of the hematoma range; (b) the speed of reduction of the arm circumference at the severest part of the hematoma; (c) the time taken for the pain to disappear; (d) the time taken for the arterial pulse to recover; (e) the time taken for the blisters to disappear; (f) the time taken for the skin color to return to normal; and (g) the time before bandage removal.

Subcutaneous hematomas were graded according to hematoma range, pain, blistering, and skin pulse, as follows: mild skin swelling with range less than one third of the forearm = level 1 or below (mild to normal); moderate skin swelling with range from one third to half of the forearm = level 1–2, occasionally with blistering and/or bruising (normal to moderate); and severe skin swelling with range greater than half of the forearm = level 2, or above if blistering, cyanosis, numbness, and weakness occur (severe).

### Statistical Analysis

Data in the present study were analyzed using statistical software SPSS 15.0. Measurement data were expressed as mean ± standard deviation (x ± SD). Count data were expressed as percentages (%). A test of normality was conducted using the *W*-test, and homogeneity of variance was tested using the *F*-test. An intergroup comparison was conducted using a *t*-test. Non-normally distributed means of multiple samples or normally distributed means of multiple samples with heterogeneity of variance were compared using a non-parametric test. Counting data were compared using a Chi-square test. The correlation was tested using logistic and Cox regression analysis, and a survival analysis curve was drawn. *P* < 0.05 was considered statistically significant.

## Results

### General Data

A total of 60 patients with moderate or severe forearm hematoma following transradial coronary intervention were enrolled in this study and randomly divided into two groups, namely, an Idealast-haft elastic bandage group and a control group (for each group, *n* = 30). The differences in general data, including age, gender, height, weight, smoking history, hypertension, diabetes, red blood cell count, hemoglobin level, platelet count, activated partial thromboplastin time, activated clotting time, levels of epidermal growth factor receptor, aspartate aminotransferase, alanine aminotransferase, antiplatelet drugs, and intraoperative heparin dosage between the two groups were not statistically significant (*P* > 0.05), as shown in Tables 2–4.

**Table 2:** Comparison of clinical baseline characteristics and laboratory test results between the two groups.

**Index**	**Idealast-Haft elastic bandage group**	**Nylexorgrip elastic bandage group**	***t***/**X^2^**	***P***
**General situation**				
Male	17/13	16/14	0.067	0.80
Age	61.97 ± 7.91	65.73 ± 8.73	−1.75	0.08
Body mass index (kg/m^2^)	26.21 ± 5.08	25.75 ± 4.68	0.36	0.72
Smoking history	14/30	12/30	0.27	0.60
Hypertension	20/30	21/30	0.08	0.78
Diabetes	10/30	9/30	0.08	0.78
Cerebral vascular disease	8/30	6/30	0.37	0.54
Atrial fibrillation	2/30	4/30	0.74	0.38
**Test results**				
Red blood cell (*10^12^/L)	4.54 ± 0.40	4.45 ± 0.56	0.75	0.46
White blood cell (g/L)	6.49 ± 1.60	6.41 ± 1.71	0.18	0.86
Platelet count (*10^9^/L)	205.45 ± 62.07	227.80 ± 57.59	−1.45	0.15
APTT (s)	36.88 ± 3.60	35.84 ± 4.05	1.05	0.30
INR (INR)	0.93 ± 0.18	0.96 ± 0.08	−0.75	0.45
eGFR (%)	91.20 ± 17.60	84.88 ± 18.90	1.34	0.19
Creatinine (umol/l)	70.36 ± 42.27	71.63 ± 16.64	−0.24	0.81
Urea nitrogen (mmol/L)	5.74 ± 1.99	5.68 ± 1.29	1.70	0.10
Alanine aminotransferase (U/L)	20.23 ± 11.42	25.57 ± 19.98	−1.27	0.21
Glutamic oxaloacetylase (U/L)	22.20 ± 11.26	26.13 ± 11.17	−1.36	0.18
Triglyceride (mmol/L)	1.71 ± 0.59	1.87 ± 1.39	−0.57	0.57
Total cholesterol (mmol/L)	4.29 ± 0.87	4.66 ± 0.83	−1.68	0.10
HGB (g/L)	135.60 ± 13.31	133.20 ± 11.29	0.75	0.46
Low density lipoprotein (mmol/L)	3.05 ± 0.57	3.27 ± 0.78	−1.23	0.22
**Echocardiography**				
LA (mm)	35.83 ± 4.36	33.00 ± 4.53	0.73	0.47
LV (mm)	47.77 ± 3.61	48.70 ± 4.14	0.93	0.36
IVS (mm)	9.87 ± 1.11	9.80 ± 1.19	0.23	0.82
LVEF (%)	60.27 ± 7.26	57.10 ± 9.38	1.46	0.15

*LVEF is left ventricular ejection fraction; IVS is interventricular septal thickness; La is left atrial transverse diameter; LV is left ventricular transverse diameter*.

**Table 3:** Diagnosis and drug treatment.

**Index**	**Idealast-Haft elastic bandage group**	**Nylexorgrip elastic bandage group**	**X^**2**^**	***P***
**Clinical diagnosis**				
STEMI	3	2	0.22	0.64
NSTEMI	2	2	0	1
UAP	17	16	0.07	0.80
AP	8	10	0.32	0.57
**Antithrombotic drugs**				
Aspirin	28	27	0.22	0.64
Clopidogrel	17	19	0.28	0.64
Ticagrelor	1	2	0.35	0.55
Tirofiban	2	1	0.35	0.55
Intraoperative heparin	16	18	0.27	0.60
Beta blocker	14	13	0.07	0.80
ACEI/ARB	8	9	0.54	0.47
Nitrate esters	13	15	0.27	0.61
Statins	21	20	0.08	0.78
Diuretic	2	3	0.22	0.64

*STEMI was acute ST-segment elevation myocardial infarction. NSTEMI was acute non-ST-segment elevation myocardial infarction. UAP was unstable angina pectoris. AP is stable angina pectoris*.

**Table 4:** Results of coronary angiography (number of cases, %).

**Index**	**Idealast-Haft elastic bandage group**	**Nylexorgrip elastic bandage group**	**X^**2**^**	***P***
LM	3	2	0.22	0.64
LAD	15	16	0.07	0.80
CTO	2	1	0.35	0.55
ISR	3	4	0.17	0.68
OS	2	3	0.22	0.64
Type C lesion	2	3	0.22	0.64
Three-vessel disease	5	6	0.11	0.74
PCI	16	15	0.01	0.60
CABG	1	2	0.35	0.35
Complete revascularization	13	14	0.07	0.80

*LM is the left trunk; LAD was the left anterior descending branch; CTO was chronic occlusive disease; ISR restenosis; PCI was used for coronary stenting. CABG is a coronary artery bypass graft*.

### Improvement of Forearm Hematoma

**Figure 1:**
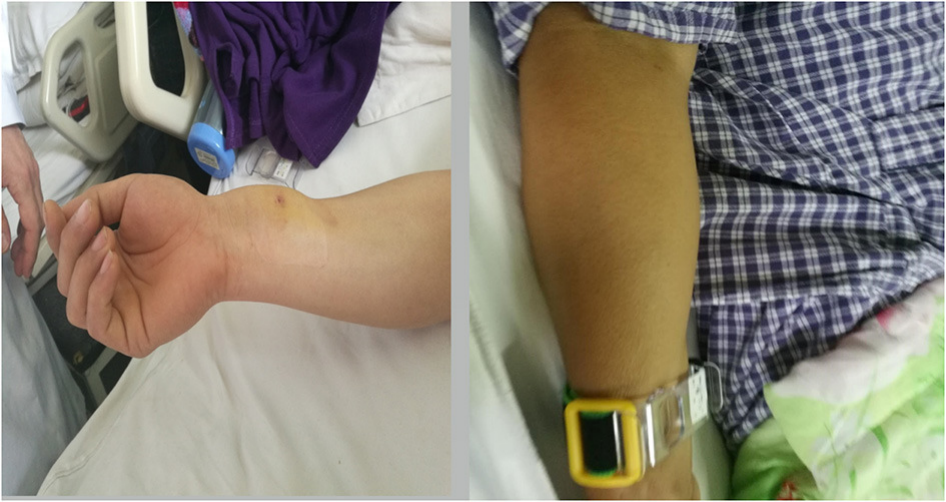
Mild hematoma.

**Figure 2:**
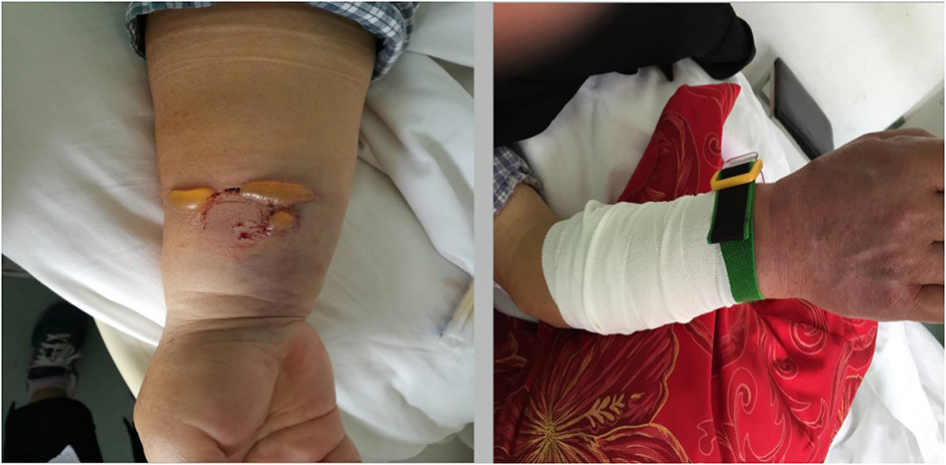
Moderate hematoma.

**Figure 3:**
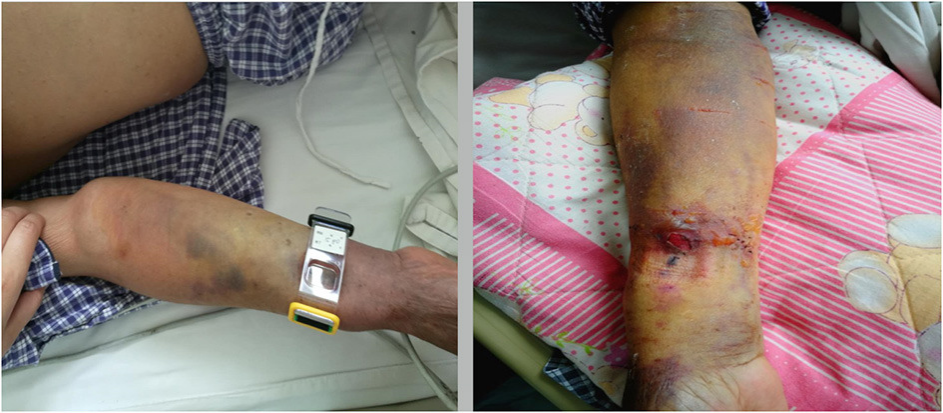
Severe hematoma.

**Figure 4:**
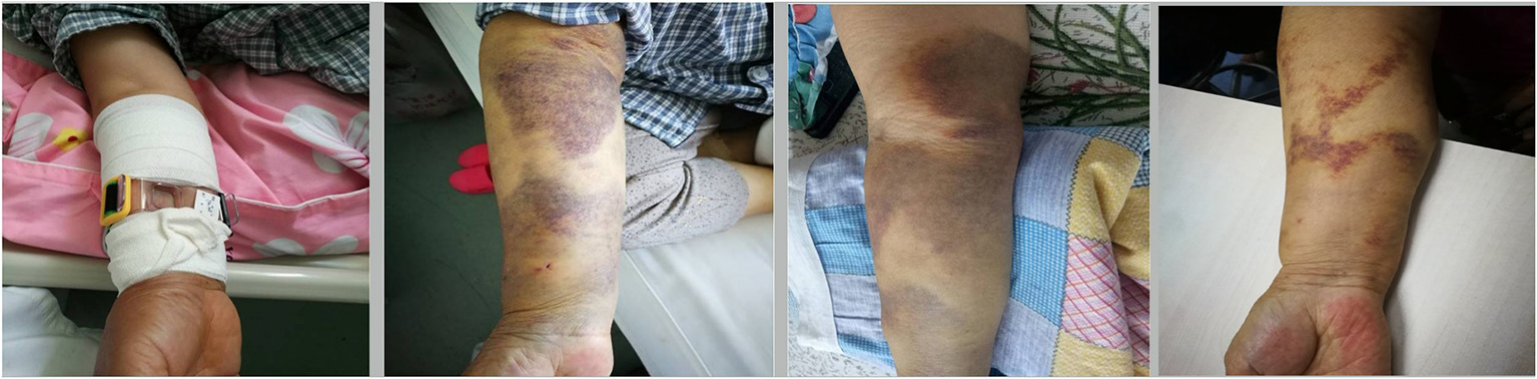
Continuous observation of moderate hematoma.

**Figure 5:**
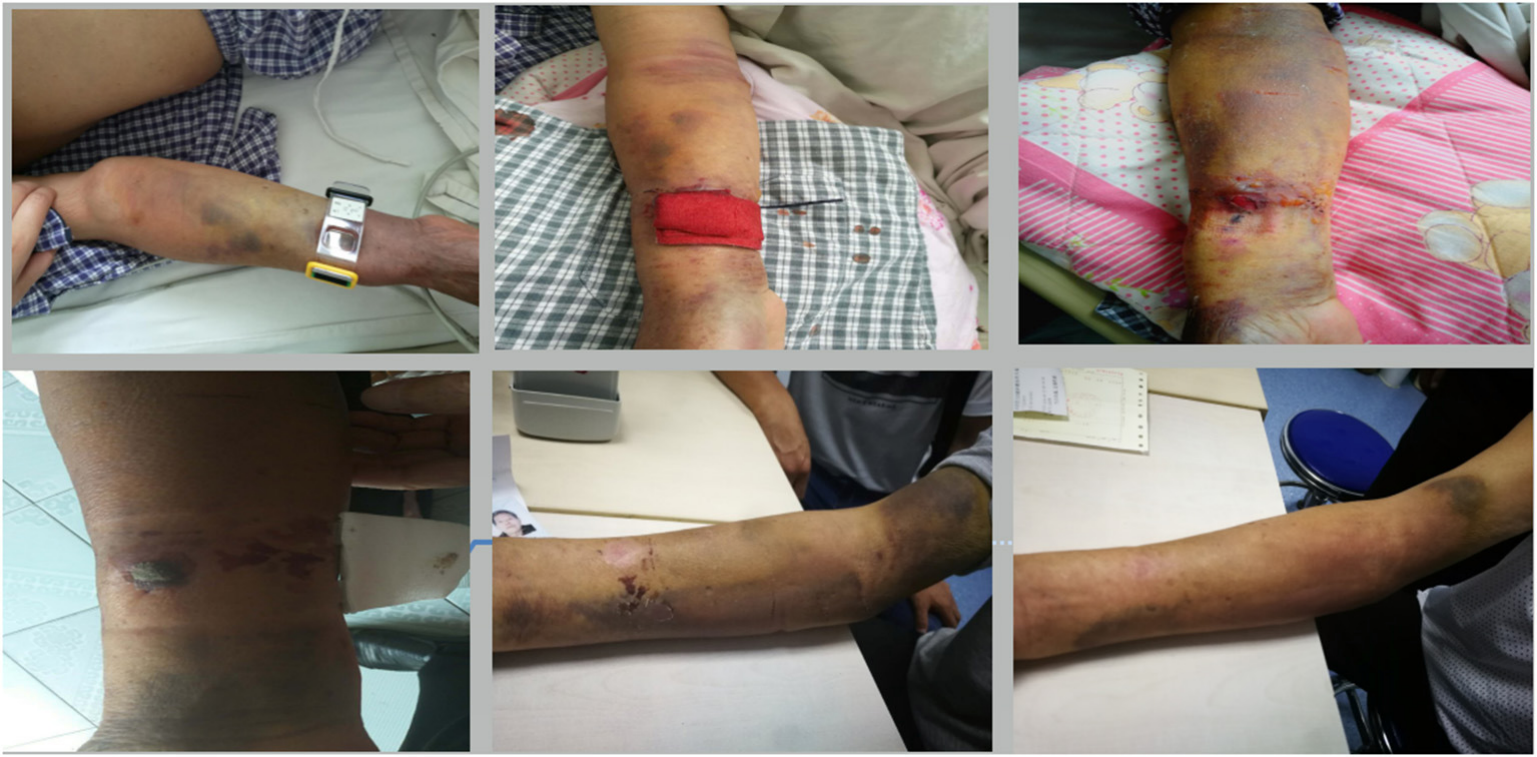
Continuous observation of severe hematoma.

The status of the forearm hematoma (including the speed of reduction of the hematoma range, the speed of reduction of the arm circumference at the severest part of the hematoma, the time taken for pain to disappear, the time taken for the arterial pulse to recover, the time taken for blisters to disappear, the time taken for the skin color to return to normal, and the time before bandage removal) were significantly better in the Idealast-haft elastic bandage group than in the control group (*P* < 0.05). Brachial artery thrombosis with severe tension hematoma occurred in one patient in the control group after the discontinuation of antithrombotic drugs, and the patient was forced to undergo a surgical incision and thrombectomy, as shown in Figures 1–5. The details are presented in Table 5.

**Table 5:** Improvements in the forearm hematoma.

**Group**	**Idealast-Haft elastic bandage group**	**Nylexorgrip elastic bandage group**	***P***
Number of cases	30	30	<0.01
Hematoma reduced by half (t)	42.47 ± 4.32	49.33 ± 4.94	<0.01
Pain disappeared (D)	23.4 ± 10.48	31.53 ± 4.85	<0.01
Arterial pulsation recovery (D)	1.7 ± 0.70	2.31 ± 0.72	<0.01
Vesicle disappeared (D)	1.43 ± 1.11	4.13 ± 1.22	<0.01
Normal skin color (D)	28.83 ± 12.50	52.23 ± 9.07	<0.01
Remove bandage (D)	1.67 ± 0.88	4.70 ± 0.98	<0.01

## Discussion

We found that the time taken for pain disappearance, arterial pulse recovery, blister disappearance, skin color recovery, and compression hemostasis were significantly shorter in the Idealast-haft elastic bandage group than in the control group, and the differences were statistically significant (*P* < 0.05). The hematoma range and arm circumference at the severest part of the hematoma decreased more rapidly in the observation group than in the control group; again, the differences were statistically significant (*P* < 0.05). The Idealast-haft elastic bandage should therefore be used for patients with forearm hematoma following transradial coronary intervention as it is more effective than the Nylexorgrip elastic bandage.

For forearm hematoma, prevention is more important than treatment. The intent of our center is to focus on prevention through the following means. (1) Preoperative prevention: The modified Allen's test is the most convenient preoperative evaluation method, and is routine in most centers. A finger oxygen saturation test and B ultrasound can also be chosen to monitor radial artery blood flow. (2) Intraoperative prevention: The occurrence of forearm hematoma is affected by the skill of the operator, the condition of the brachial artery, and the use of antithrombotic drugs before and during the operation. During the operation, the number of punctures should be minimized, and the operation should be gentle; when the guidewire is difficult to ascend, radial arteriography should be performed immediately, and a smooth guidewire with hydrophilic coating should be used. (3) Postoperative prevention: If the puncture site is not properly compressed after the operation, blood can leak into the subcutaneous tissue of the forearm. The presence and the condition of the hematoma in the position of the pressure apparatus and the forearm should be observed, particularly at three time points, namely, by the surgeon when the operation is completed and when the instruments are removed, by the assistant after the surgeon steps down, and by the nurse after the patient returns to the ward. Meanwhile, attention should be paid to the aggravation of ischemia symptoms such as pain, numbness, and abnormal activity, and the pressure of the elastic bandaging should be reduced every 2 h. Elastic bandaging that is too tight will lead to venous blood reflux obstruction; if this continues over a long period of time, it may slow the blood flow in the hand, and the nerve endings may become dull due to the lack of oxygen, leading to numbness (10–12). Pain at the compression site is mainly due to compression of the nerve (13). If the above situation occurs, the bandage should be released immediately to aid decompression (14). The Idealast-haft elastic bandage comprises of polyester fiber (45%), cotton (42%), and fiber adhesive (13%), with an adhesive coating made of natural rubber. The main materials of the Nylexorgrip elastic bandage are fiber adhesive (46%), face glue (42%), and elastic fiber (9%); it is covered with latex particles. These two types of elastic bandage differ somewhat in their materials, and the respective manufacturer of each has its peculiar craft.

Cold compression with an ice bag at the swelling area can shrink blood vessels and slow down blood flow, thereby relieving pain, bleeding, and hematoma. In a study involving a total of 38 patients with forearm hematoma following coronary intervention, Berner et al. compared the results of injecting dehydrating agents in the hand dorsal vein between the affected and the non-affected sides. They found that injection in the hand dorsal vein of the affected side could relieve pain faster, promote better edema regression, and reduce the occurrence of tension blisters (15). Jonker et al. compared the curative effects of tension hematoma on the anterior wall following coronary intervention of hand compression, elastic bandage compression alone, and bandage compression combined with dehydration. The results revealed that bandage compression combined with dehydration had a better clinical effect (16). When the patient has numbness, inactivity, and finger dyskinesia, clinicians should be alert to osteofascial compartment syndrome and, if necessary, puncture or surgical incision and drainage should be performed. Sphygmomanometer cuff compression is relatively convenient and simple to use, and is therefore a method worthy of tentative clinical application. Many centers currently use this method (17, 18).

The current treatment in most medical centers is local compression hemostasis using a pressure apparatus and an elastic bandage. When the radial artery is punctured, the puncture needle is inserted at 30°-45° to the skin. Therefore, when a forearm hematoma occurs in our center, first, the position of the pressure apparatus is checked and adjusted to ensure that it is 0.5 cm above the subcutaneous entry point (near the heart end), which is the center point for local compression hemostasis. However, sometimes blood spreads to the surrounding area along the tissue gap, resulting in subcutaneous bleeding and forearm hematoma. In such cases, our center mostly uses elastic bandages to wholly wrap the hematoma site and moderately compress it. The pressure of the elastic bandage should be maintained within the levels in which the pulsation of the radial artery of the affected limb can be felt, and patients should be monitored for the presence of pain, numbness, abnormal activity, and other ischemic symptoms. If these occur, the bandage should be released and the pressure should be reduced (19). At present, widely used elastic bandages include the German Idealast-haft bandage and the French Nylexorgrip bandage. The hemostatic effect will differ between bandages due to the different materials used, the manufacturing processes, tension, and other factors. The results of our study suggest that the Idealast-haft bandage has better curative effects in treating forearm hematomas than the Nylexorgrip bandage.

In this study, severe tension hematoma of the forearm occurred in one patient following the interventional operation, necessitating the use of general antithrombotic drugs, embolization of the brachial artery of the right upper limb, surgical incision, and embolectomy. The reason may be that the subcutaneous hematoma was found late and could not be treated in time; in addition, once it was discovered, we overemphasized compression with elastic bandaging at the hematoma and ignored timely hemostasis at the puncture point. In other words, although elastic bandage tension has the effect of local compression hemostasis, it must be carried out on the local puncture point. The discontinuation of antithrombotic drugs also indirectly induced brachial artery embolism. Therefore, the decision of whether to discontinue antithrombotic drugs should be individualized, and the risk of bleeding and thrombosis in the upper limbs and coronary artery should be weighed repeatedly (20).

There are some limitations to this study. First, although this was a randomized controlled trial, it was not blinded; therefore, a risk of bias remains. Second, this was a single-center clinical trial and the sample size is small. A multicenter clinical trial with a larger sample size is needed.

## Conclusion

Although prevention is more important than treatment for forearm hematoma, the results of this study suggest that, when a forearm hematoma occurs, the Idealast-haft elastic bandage has a better compressive effect than the Nylexorgrip bandage and is, therefore, worthy of clinical popularization.

## Data Availability Statement

The original contributions presented in the study are included in the article/supplementary material, further inquiries can be directed to the corresponding author.

## Ethics Statement

The studies involving human participants were reviewed and approved by Ethics Committee of Langfang people hospital. The patients/participants provided their written informed consent to participate in this study. Written informed consent was obtained from the individuals for the publication of any potentially identifiable images or data included in this article.

## Author Contributions

LA and W-lD conceptualized and designed the study, drafted the initial manuscript, and reviewed and revised the manuscript. LA, X-NY, and C-YZ collected data, carried out the initial analyses, and reviewed and revised the manuscript. Z-MX coordinated, supervised data collection, and critically reviewed the manuscript for important intellectual content. All authors contributed to the article and approved the submitted version.

## Funding

This study was funded by Scientific Research project of Langfang Science and Technology Bureau in 2016 (project number: 2016013019). The funding body had no role in the design of the study and collection, analysis, and interpretation of data and in writing the manuscript.

## Conflict of Interest

The authors declare that the research was conducted in the absence of any commercial or financial relationships that could be construed as a potential conflict of interest.

## Publisher's Note

All claims expressed in this article are solely those of the authors and do not necessarily represent those of their affiliated organizations, or those of the publisher, the editors and the reviewers. Any product that may be evaluated in this article, or claim that may be made by its manufacturer, is not guaranteed or endorsed by the publisher.
